# Capturing nystagmus in the emergency room: posterior circulation stroke versus acute vestibular neuritis

**DOI:** 10.1007/s00415-022-11202-y

**Published:** 2022-07-18

**Authors:** B. Nham, G. Akdal, A. S. Young, P. Özçelik, T. Tanrıverdizade, R. T. Ala, A. P. Bradshaw, C. Wang, S. Men, B. F. Giarola, D. A. Black, E. O. Thompson, G. M. Halmagyi, M. S. Welgampola

**Affiliations:** 1 Neurology Department, Royal Prince Alfred Hospital, Sydney, Australia; 2 Faculty of Medicine and Health, Institute of Clinical Neurosciences, Royal Prince Alfred Hospital, Central Clinical School, University of Sydney, Sydney, Australia; 3 Faculty of Medicine, Department of Neurology, Dokuz Eylül University, İzmir, Turkey; 4 Department of Neuroscience, Institute of Health Sciences, Dokuz Eylül University, İzmir, Turkey; 5 Faculty of Medicine, Department of Radiology, Dokuz Eylül University, İzmir, Turkey; 6 Department of Radiology, Royal Prince Alfred Hospital, Sydney, Australia; 7 Faculty of Medicine and Health, University of Sydney, Sydney, Australia

**Keywords:** Nystagmus, Vertigo, Stroke, Vestibular neuritis, Emergency medicine

## Abstract

**Objectives:**

To compare acute nystagmus characteristics of posterior circulation stroke (PCS) and acute vestibular neuritis (AVN) in the emergency room (ER) within 24 h of presentation.

**Methods:**

ER-based video-nystagmography **(**VNG) was conducted, recording ictal nystagmus in 101 patients with PCS (on imaging) and 104 patients with AVN, diagnosed on accepted clinical and vestibular test criteria.

**Results:**

Patients with stroke in the brainstem (38/101, affecting midbrain (*n* = 7), pons (*n* = 19), and medulla (*n* = 12)), cerebellum (31/101), both (15/101) or other locations (17/101) were recruited. Common PCS territories included posterior-inferior-cerebellar-artery (41/101), pontine perforators (18/101), multiple-territories (17/101) and anterior-inferior-cerebellar-artery (7/101). In PCS, 44/101 patients had no spontaneous nystagmus. Remaining PCS patients had primary position horizontal (44/101), vertical (8/101) and torsional (5/101) nystagmus. Horizontal nystagmus was 50% ipsiversive and 50% contraversive in lateralised PCS. Most PCS patients with horizontal nystagmus (28/44) had unidirectional “peripheral-appearing” nystagmus. 32/101 of PCS patients had gaze-evoked nystagmus. AVN affected the superior, inferior or both divisions of the vestibular nerve in 55/104, 4/104 and 45/104. Most (102/104) had primary position horizontal nystagmus; none had gaze-evoked nystagmus. Two inferior VN patients had contraversive torsional-downbeat nystagmus. Horizontal nystagmus with SPV ≥ 5.8 °/s separated AVN from PCS with sensitivity and specificity of 91.2% and 83.0%. Absent nystagmus, gaze-evoked nystagmus, and vertical-torsional nystagmus were highly specific for PCS (100%, 100% and 98.1%).

**Conclusion:**

Nystagmus is often absent in PCS and always present in AVN. Unidirectional ‘peripheral-appearing’ horizontal nystagmus can be seen in PCS. ER-based VNG nystagmus assessment could provide useful diagnostic information when separating PCS from AVN.

**Supplementary Information:**

The online version contains supplementary material available at 10.1007/s00415-022-11202-y.

## Introduction

Stroke accounts for 4–12% of vertigo and imbalance presentations to the emergency room (ER) and is often misdiagnosed [1–3]. Given the availability of reperfusion strategies, early diagnosis and treatment of posterior circulation stroke (PCS) can reduce patient mortality, morbidity and prevent future cerebrovascular events [4].

The HINTS (Head Impulse, Nystagmus, Tests for Skew) algorithm is an excellent bedside tool, that outperforms magnetic resonance imaging (MRI) in diagnosing PCS but relies on the competence of the clinician detecting nystagmus by visual inspection [5, 6]. Capturing and measuring nystagmus is a potentially useful adjunctive diagnostic technique that does not rely on specialty expertise, and can assist in increasing the diagnostic accuracy of detecting PCS in the ER [7].

Peripheral vestibular nystagmus from acute vestibular neuritis (AVN), is expected to be unidirectionally horizontal-torsional in straight-ahead gaze (primary position), suppressed by visual fixation and obey Alexander’s law [8]. Pathologic gaze-evoked nystagmus occurs in central aetiologies [8]. It can appear with or without nystagmus in the primary position but has a gaze-dependent reversal of nystagmus direction (e.g., left beating nystagmus in left gaze and right-beating in right gaze).

Video-nystagmography (VNG) is a useful method of nystagmus assessment. It enables the removal of nystagmus suppression by visual fixation, enhancing nystagmus of peripheral causes such as AVN, more than nystagmus of central causes such as PCS, enabling easier observation by the examiner [9]. In the ER, VNG increases the capture rate of acute nystagmus which otherwise would be missed by the naked eye [10].

The nystagmus characteristics in PCS vary according to the vascular territories involved and the location of the resulting stroke. Posterior inferior cerebellar artery (PICA) strokes cause either ipsiversive or contraversive horizontal, up-beating or torsional down-beating nystagmus and gaze-evoked nystagmus [11–13] Anterior inferior cerebellar artery (AICA) strokes predominantly cause contraversive horizontal peripheral type nystagmus, gaze-evoked nystagmus and sometimes Bruns nystagmus [14–16] whereas superior cerebellar artery (SCA) strokes cause ipsiversive horizontal nystagmus or gaze-evoked nystagmus [17]. Pontine perforator involvement in basilar artery stroke seldom causes nystagmus but if present can be vertical [18]. However, these nystagmus descriptions were not recorded within the ER and hence may be not truly reflective of the acute nystagmus patterns of PCS seen by frontline emergency and neurology physicians.

The aim of our study was to undertake bedside event monitoring using VNG in the ER, in patients presenting with the acute vestibular syndrome (AVS) and compare nystagmus characteristics in PCS with AVN.

## Methods

**Fig. 1:**
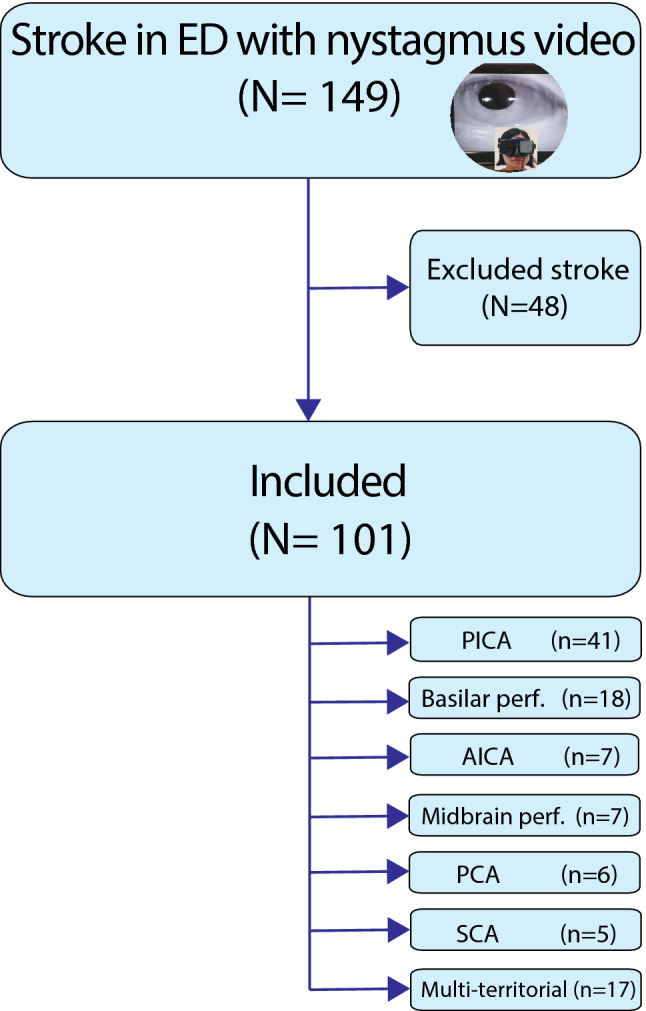
Flowchart. Flowchart of participants

Recruitment took place between Jan 2016 and December 2020 for 149 patients with radiologically confirmed PCS presenting with acute vertigo and/or imbalance to the emergency departments at two sites (Royal Prince Alfred Hospital, Sydney, Australia and Dokuz Eylul University Hospital, Izmir, Turkey). Each patient underwent ictal nystagmus recording with VNG in ER. VNG recordings were made with the patient in the upright position. Forty-eight patients who had VNG recording more than one day after ER presentation were excluded from this study (Fig. 1). Patients presenting to ER with AVS who were diagnosed with AVN were studied for comparison. AVN was diagnosed in patients with (1) isolated spontaneous vertigo lasting 24 h or longer, with (2) abnormal clinical horizontal head impulse test to only one side or an abnormal video head impulse consistent with superior, inferior or pan-neuritis and (3) no reported hearing loss. One hundred and one PCS and 104 AVN patients had VNG within 24 h of presentation. Although nystagmus was recorded on day one in ER, the median time from symptom onset to VNG for PCS was one day (mean 1.5 ± 2.4, range 0–13 days). Median time from symptom onset to VNG in AVN was also one day (mean 1.1 ± 1.5, range 0–6 days).

Consenting patients had VNG recording with our custom-built portable monocular video nystagmus goggles [10, 19]. With the patient sitting upright, nystagmus was recorded in the primary position without visual fixation. Nystagmus was then recorded while gazing to the left and the right whilst upright, followed by a recording in the left and then right Dix-Hallpike positions. Videos were recorded in 720 × 576 pixel frames at 30 Hz and analysed using custom-written pupil-tracker software utilising threshold-based ellipse fitting and calibration on a LabVIEW (National Instruments, Austin, Texas) platform [20]. Nystagmus was analysed in horizontal and vertical planes, and nystagmus slow phase velocity (SPV) was measured in degrees per second (°/s) and plotted as a function of time. Torsional nystagmus was observed but not measured. Whilst all patients had VNG nystagmus recordings without visual fixation, a proportion had then had recordings with visual fixation immediately afterwards. All nystagmus results, reported in this study are recorded without visual fixation unless otherwise specified.

PCS location in each patient was initially determined by the reporting radiologist based on relevant MRI-DWI or CT sequences and then reviewed by a neuroradiologist author (EOT, SM, BG) blinded to the initial report. Stroke vascular territory was determined by the neuroradiologist, according to a published atlas [21].

### Statistical analysis

Statistics were performed using SPSS Statistics for Windows, Version 26.0 (Armonk, NY). The relationship between binary outcomes (e.g., normal and abnormal) for various test combinations were explored using cross-tabulations with Chi-square tests or Fisher’s exact test for analysis. For the non-parametric SPV data, Mann–Whitney *U* Tests were used. Significance was determined at the 5% level of significance. Sensitivity and specificity were calculated using ROC curves with Youden’s index for threshold selection.

### Ethics and consent

This study received local ethics committee approval for the use of human participants and written informed consent was obtained from all participants in accordance with the Helsinki Declaration of 1975.

## Data availability statement

Data not published within the article is available and anonymized data will be shared by request from any qualified investigator.

## Results

We report 101 patients with PCS (93 ischemic, 8 haemorrhagic) whose neurological examination included VNG recordings in ER on the day of presentation. PCSs were shown on MRI in 88 and CT in 11. For comparison 104 consecutive patients with AVN presenting to ER over the same overlapping 24-month recruitment period were also studied.

Presenting symptoms and demographics are summarised in Table 1. Most (70/104) PCS patients and all AVN patients presented with AVS. PCS patients were significantly older (*p* < 0.001) than AVN patients and more likely to have additional neurological abnormalities, as well as more than two vascular risk factors such as hypertension, diabetes, dyslipidaemia (*p* < 0.001).

**Table 1:** Patient demographics and clinical characteristics

	Stroke (*n* = 101)	Vestibular neuritis (*n* = 104)	*p*-value
Age, mean years (SD)	67.0 (12.3)	56.9 (17.0)	** < 0.001**
Female, *n* (%)	33 (32.7)	45 (43.2)	0.118
*Vascular risk factors, n (%)*			
Hypertension	68 (67.3)	31 (29.8)	** < 0.001**
Diabetes	30 (29.7)	5 (4.8)	** < 0.001**
Hyperlipidaemia	50 (49.5)	20 (19.2)	** < 0.001**
Atrial fibrillation	16 (15.8)	5 (4.8)	0.009
Smoking	22 (21.8)	11 (10.6)	0.029
Arterial dissection (previous or recent), *n* (%)	9 (8.9)	1 (1.0)	0.008
Haemorrhagic stroke, *n* (%)	8 (7.9)	N/A	N/A
Ischemic stroke, *n* (%)	93 (92.1)	N/A	N/A
*Presenting vertigo syndrome, n (%)*			
Acute vestibular syndrome	70 (69.3)	104 (100)	** < 0.001**
Acute transient vestibular syndrome	10 (9.9)	0	**0.001**
Acute/Subacute ataxia	17 (16.8)	0	** < 0.001**
Episodic spontaneous vertigo	1 (1.0)	0	0.493
Episodic positional vertigo	0	0	N/A
Other	3 (3.0)	0	0.118
Gaze evoked nystagmus, *n* (%)	32 (31.7)	0 (0)	** < 0.001**
*Laterality of stroke/neuritis, n %*			
Right sided	39 (38.6)	58 (55.7)	0.014
Left sided	46 (45.5)	46 (44.2)	0.927
Multiple or midline	16 (15.9)		N/A
*Nystagmus (dominant SPV), n (%)*			
Nil	44 (43.5)	0	** < 0.001**
Horizontal	44 (43.5)	102 (98.1)	** < 0.001**
Vertical	8 (8.0)	0	0.003
Torsional	5 (5.0)	2 (1.9)	0.445
*Additional neurological signs, n (%)*			
Weakness	22 (21.8%)	0	** < 0.001**
Hemisensory loss	10 (9.9%)	0	** < 0.001**
Dysarthria	24 (23.7%)	0	** < 0.001**
Limb ataxia or incoordination	30 (29.7%)	3 (2.9%)	** < 0.001**
Truncal ataxia or severe gait ataxia	60 (59.4%)	6 (5.8%)	** < 0.001**
Diplopia	20 (19.8%)	0	** < 0.001**
Acute hearing loss	2 (2.0%)	0	0.118

### Overall nystagmus characteristics in PCS and AVN

PCS patients had predominantly horizontal nystagmus (44/101) or vertical nystagmus (8/101) or torsional nystagmus (5/101). Of the 104 PCS patients, 44 had no nystagmus. Of the 38 patients with lateralised PCS and horizontal nystagmus, 14 had ipsiversive and 14 had contraversive nystagmus (as defined by the nystagmus fast phase).

**Fig. 2:**
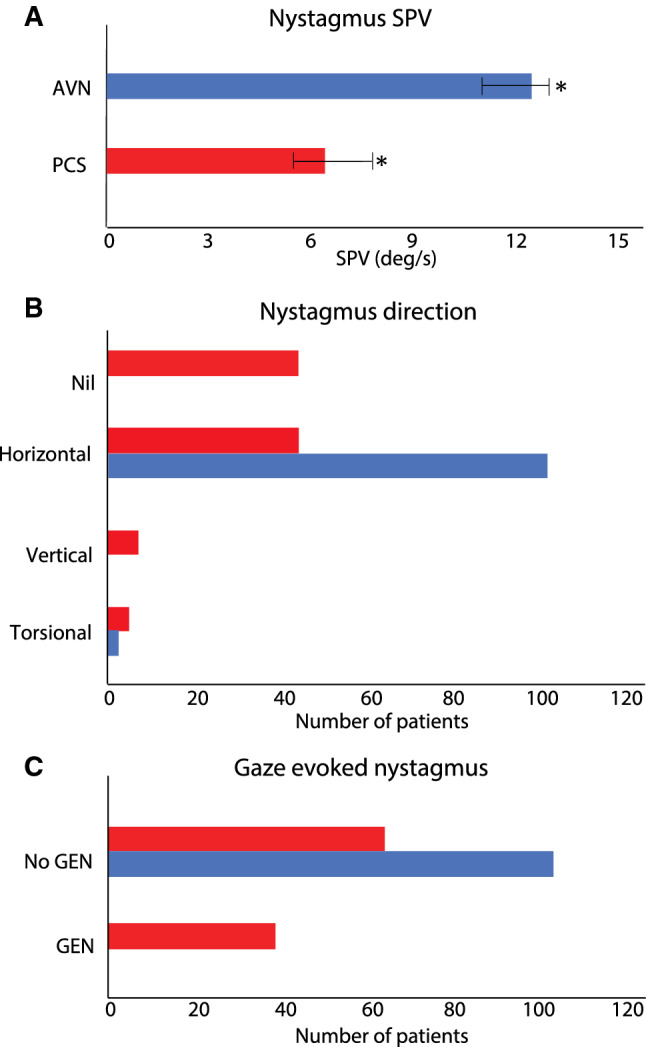
Comparison of PCS and AVN nystagmus characteristics. Red bars denote PCS and blue bars denote AVN. In Fig. 2A, Nystagmus SPV was significantly faster in AVN (11.7 ± 6.5°/s) than PCS (6.1 ± 4.7°/s) (*p* < 0.001) Nystagmus direction was almost always horizontal (98.1%) in AVN whereas PCS patients had predominantly no nystagmus (i.e., absence of nystagmus) or horizontal nystagmus with a small proportion of vertical and torsional nystagmus (Fig. 2B) No patients with AVN had bidirectional GEN whilst 31.7% of PCS patients had bidirectional GEN (Fig. 2C). *AVN* acute vestibular neuritis, *deg/s* degrees per second, *GEN*  gaze-evoked nystagmus, *PCS*  posterior circulation stroke, *SPV*  slow phase velocity]

Only 16/44 (36.3%) of PCS patients with primary-position horizontal nystagmus also had direction changing ‘central-appearing’, gaze-evoked nystagmus. The mean horizontal SPV in PCS (3.0 ± 4.6 °/s; range 1.3–26.5°/s) was significantly lower than in AVN patients (12.3 ± 7.2 °/s; range 2.1–42.9 °/s) (Fig. 2A, *p* < 0.001). Unidirectional horizontal nystagmus with SPV ≥ 5.8°/s separated PCS from AVN with a sensitivity of 91.2% and specificity of 83.0%. Horizontal nystagmus with SPV ≤ 5.6 °/s separated PCS from AVN with a specificity of 90.2% and sensitivity of 61.4%.

AVN diagnosis was based on history (acute spontaneous vertigo and/or imbalance), examination (clinically positive bedside head impulse test) and confirmatory vestibular testing. Vestibular testing comprised of video head impulses to assess all three semicircular canals and vestibular-evoked myogenic potentials to assess the utricle and sacculus. Cases were classified as pan-, superior- or inferior- vestibular neuritis (43.3%, 52.9%, 3.8%).

Unlike in PCS, nystagmus characteristics in AVN were distinctive: nearly all AVN patients (98.1%) had contraversive, unidirectional horizontal nystagmus (Fig. 2B). Two patients with AVN had contraversive, downbeat-torsional nystagmus consistent with inferior neuritis.

PCS patients were more likely than AVN patients to have pathologic gaze-evoked nystagmus (31.4% of PCS, 0% of AVN, Fig. 2C, *p* < 0.001).

Three characteristics were highly specific for PCS: (1) direction-changing gaze-evoked nystagmus (100%), (2) primary position vertical or torsional nystagmus (98.1%) AND (3) absence of nystagmus even in the absence of visual fixation (100%).

### Nystagmus by location and vascular territory

**Fig. 3:**
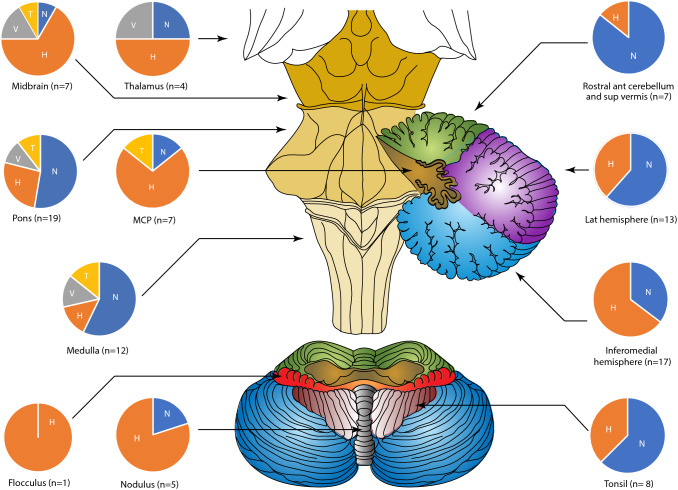
Breakdown of nystagmus characteristics by stroke location. [Pie chat abbreviations: *H*  horizontal nystagmus (orange), *N*  nil nystagmus (blue), *T*  torsional nystagmus (yellow), *V*  vertical nystagmus (grey)] *ant*  anterior, *MCP*  middle cerebellar peduncle, *lat*  lateral, *sup*  superior][Colours of anatomical locations: Green = rostral anterior cerebellum and superior vermis, Purple = lateral cerebellar hemisphere, Blue = Inferomedial cerebellum, Dark brown = Middle cerebellar peduncle, Red = Flocculus, Maroon = Tonsil, Grey = Nodulus]

**Fig. 4:**
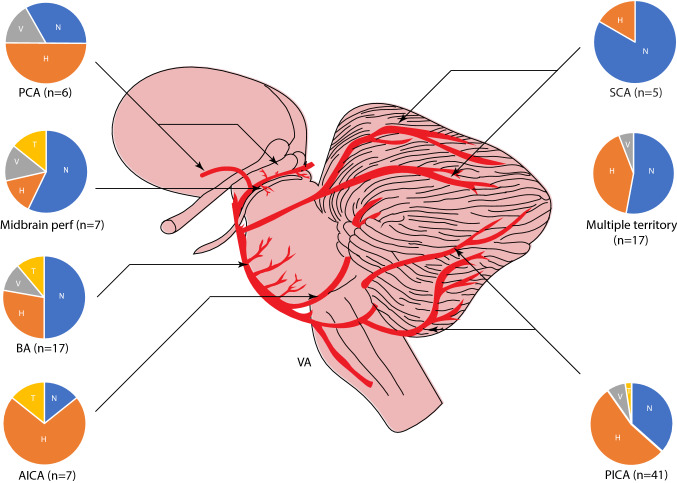
Nystagmus characteristics by stroke vascular territory. [Pie chart abbreviations: *H*  horizontal nystagmus (orange), *N*  nil nystagmus (blue), *T*  torsional nystagmus (yellow), *V*  vertical nystagmus (grey)] *AICA*  anterior inferior cerebellar artery, *BA*  basilar artery perforators, *PCA*  posterior cerebral artery, *PICA*  posterior inferior cerebellar artery, *SCA*  superior cerebellar artery, *VA*  vertebral artery]

We summarize the most common anatomic locations and vascular territories of PCS in descending order (Table 2). Nystagmus features of PCS by anatomical location and vascular territory are shown in Figs. 3 and 4.

**Table 2:** Vascular territories and Anatomical location of PCS

PCS	(*n* = 101)
Vascular territory	
PICA	41
Basilar perforators	18
Multi-territorial	17
AICA	7
Midbrain perforators	7
PCA	6
SCA	5
Brainstem alone (*n* =)	38
Midbrain	7
Pons	19
Medulla	12
Cerebellum alone (*n* =)	31
Lateral hemispheres involved	13
Inferomedial hemisphere involved	17
Superior vermis involved	7
Tonsil involved	8
Uvula/nodulus involved	5
Flocculus involved	1
Brainstem and cerebellum (*n* =)	15
MCP involved	7
Thalamus (*n* =)	4
Multiple (*n* =)	11
Multiple posterior territory infarcts	6
Anterior and posterior territory infarcts	5
Cortex (*n* =)	2
Occipital cortex	2

*AICA*  anterior inferior cerebellar artery, *MCP*  middle cerebellar peduncle, *PCA*  posterior cerebral artery, *PICA*  posterior inferior cerebellar artery, *PCS*  posterior circulation stroke, *SCA*  superior cerebellar artery

### Nystagmus characteristics by anatomical location

Twenty-six strokes involved more than one anatomical location in the posterior circulation. Common locations were the brainstem (*n* = 58) and cerebellum (*n* = 52).

Isolated Cerebellar strokes: Of 52 PCSs involving the cerebellum, 31 were isolated to the cerebellum and the patient either had no nystagmus (17/31) or purely horizontal nystagmus (14/31: 8 ipsiversive, 5 contraversive and 1 left-beating in a bilateral cerebellar hemispheric stroke).

Brainstem strokes: Nystagmus prevalence was high in PCSs with brainstem involvement (37/58), but no significant difference was detected compared with all PCSs (χ^2^, *p* = 0.07). Brainstem-only strokes were more likely to have vertical (χ^2^, *p* = 0.007) and torsional (χ^2^, *p* = 0.012) nystagmus compared to other locations (Fig. 3). The pons was the most common location (19/38) of brainstem-only stroke and was less likely to have horizontal nystagmus compared with other brainstem sites (χ^2^, *p* = 0.015). The medulla was more likely to have horizontal nystagmus than other brainstem sites (χ^2^, *p* = 0.003). The medulla had a higher prevalence of ictal nystagmus (χ^2^, *p* = 0.014) and gaze-evoked nystagmus (χ^2^, *p* = 0.044) compared to the other brainstem sites. Four of 7 midbrain strokes did not have nystagmus.

### Nystagmus characteristics by vascular territory

In terms of vascular territory involvement, PICA strokes were most common and SCA territory infarcts least common (Table 2).

PICA: Nystagmus was absent in 15/41 of PICA strokes (Fig. 4); 22/41 had predominantly horizontal nystagmus (11/21 ipsiversive, 10/21 contraversive, 1/21 left beating in bilateral PICA, SPV 6.1 ± 4.7 °/s) followed by torsional-vertical in 4/41 (SPV 18.7 ± 22.1 °/s). Multiple nystagmus planes (horizontal-torsional = 5, horizontal-torsional-vertical = 6) were recorded in 11 strokes. PICA infarcts including those involving the uvula, nodulus or lateral medulla were significantly more likely to have gaze-evoked nystagmus, compared to all other territories combined (χ^2^, *p* = 0.025). PICA strokes affecting the brainstem alone (PICAb) were significantly more likely to produce contralesional horizontal nystagmus compared with all other PCS with nystagmus (χ^2^, *p* = 0.04) whereas pure PICA cerebellar strokes (PICAc) were more likely to have ipsilesional horizontal nystagmus compared with all PCS with nystagmus (χ^2^, *p* = 0.025).

AICA and SCA: There were seven AICA strokes: one solely involving the brainstem, two involving the cerebellum alone and four involving both brainstem and cerebellum i.e., the cerebellar peduncles. Six of seven patients had nystagmus, five presented with horizontal nystagmus (1/5 ipsiversive, 4/5 contraversive) with a mean SPV of 5.2 ± 3.9 °/s, the remaining patient had contraversive torsional nystagmus. Two patients with AICA PCS also had acute ipsilesional hearing loss. Only one of the 5 SCA strokes showed nystagmus; this was horizontal and contraversive.

Basilar perforator: There were eighteen basilar perforator strokes—sixteen involving the pons and two involving the pontomedullary junction. No nystagmus was observed on 9/18, followed by horizontal (5/18: 3 ipsiversive, 2 contraversive), vertical (2/18: 1 upbeat, 1 downbeat) and torsional (2/18) nystagmus.

### Effect of visual fixation

Seventy-two PCS and ninety AVN patients had VNG nystagmus recordings with a visual fixation for immediate comparison with their VNG nystagmus recordings without visual fixation. Mean horizontal nystagmus SPV with fixation in PCS (1.6 ± 2.5 °/s, range 0–9.6 °/s) was significantly slower than in AVN (6.4 ± 5.2 °/s, range 0–28.3 °/s) (*p* < 0.001). There was a greater increase in nystagmus SPV with visual fixation suppression in AVN compared to PCS. Mean absolute nystagmus SPV difference between no visual fixation and fixation conditions was significantly higher in AVN (5.9 ± 4.7 °/s) compared to PCS (0.7 ± 2.3 °/s) (*p* < 0.001).

## Discussion

We found that more than half of our 104 PCS patients presenting acutely to ER with vertigo, dizziness or imbalance demonstrated ictal nystagmus on bedside VNG.

Our study draws attention to two observations:

1. Even with visual fixation removed, 34.5% of PCS patients had no nystagmus in the primary position. In contrast, all AVN patients demonstrated ictal nystagmus when visual fixation was removed. Thus, in the context of acute vestibular syndrome, the absence of nystagmus in a symptomatic patient was 100% specific for a diagnosis of PCS. This finding of a high rate of absent nystagmus is PCS, even with VNG use, is novel. Previous investigators found that 17% of all PCS presenting with AVS had no nystagmus, with 75% of a small subset of these patients with “false negative central HINTS pattern” having absent nystagmus despite video-Frenzel glasses [22].
  The neurologist could then erroneously equate the absence of nystagmus with the absence of abnormality in the patient with acute spontaneous vertigo and thereby inappropriately discharge them from ER without further investigation. In this clinical situation, as in some others, absence of evidence is not evidence of absence. Thus, we recommend that in a symptomatic patient with AVS, the absence of spontaneous nystagmus with visual fixation removed should be interpreted as a central sign within the HINTS algorithm. A full neurological examination for abnormalities such as localising brainstem deficits and truncal ataxia should be sought to strengthen any suspicion. These recommendations have already been proposed in the setting of the acute transient vestibular syndrome [23, 24]. In comparison to our cohort of predominantly AVS patients, the yield of central lesions in a separate cohort of patients with no nystagmus and imbalance was only 33% [3]. This probably reflects the prevalence of non-neurological causes of imbalance e.g., orthostatic hypotension or electrolyte disturbance.
2. Of the PCS patients, 63.6% with horizontal nystagmus appeared to have “peripheral-appearing” nystagmus i.e., they did not have direction-changing, gaze-evoked nystagmus. Primary position vertical or torsional nystagmus, considered to be indicative of central nystagmus, was not common in PCS and accounted for less than 13% of all nystagmus observations in our PCS patients. Our observations reinforce the importance of the head impulse test in differentiating PCS from AVN within the HINTS algorithm. The HINTS plus algorithm may incorrectly classify the rare cases of inferior vestibular neuritis as central and in these situations vHIT may be helpful [25].

The present study reproduced 3 previously reported anatomical correlations: (1) Gaze-evoked nystagmus occurs in PICA territory strokes – sites implicated in the literature and represented in our study (without sufficient power) include the lateral medulla, uvula and tonsil [13, 26]; (2) Ipsiversive horizontal nystagmus is common in PICAc strokes; and (3) Contraversive horizontal is common in PICAb infarcts [26]. This direction preponderance is thought to be due to the involvement of the medial vestibular nucleus in PICAc strokes and the caudal lateral vestibular nucleus in PICAb strokes [27, 28].

PCS can occur in a diverse range of locations, which are associated with different patterns of horizontal, vertical and/or torsional nystagmus in the literature (Table 3). Downbeat nystagmus has been attributed to lesions with the brainstem affecting the medial longitudinal fasciculus, which was seen in our study, or bilateral lesions within the cerebellar flocculus or paraflocculus [29–31]. Isolated unilateral flocculus infarcts are reported to cause ipsilesional horizontal nystagmus [32]. Flocculus involvement in our study demonstrated contraversive nystagmus, but this was probably because other structures were also affected. Upbeat nystagmus has been associated with any location within the brainstem, mainly in the paramedian areas and especially when the ventral tegmental tract is involved [33]. Finally, we found five strokes with torsional nystagmus attributed to lesions within the midbrain, middle cerebellar peduncle or lateral medulla. Interrupted brainstem projections from the vertical semicircular canals or involvement of mesencephalic torsional quick-phase generators may have been responsible [34–36].

**Table 3:** Anatomical-nystagmus correlation with vascular territory and lesion site

Territory	Horizontal	Vertical	Torsional	Vert-Tors	Horiz-Tors
Med PICA	medial VNc [10], ICP [12, 23], tonsil [12, 23], nodulus [12, 23], uvula [12, 23]	Nodulus (bilateral) [28]		Superior VNc [25, 26]	ICP [12, 23]
Lat PICA	Lateral medulla [8, 33], Caudal lateral VNc [24, 26]	Caudal medulla [23]	Lateral medulla [32]	Lateral medulla [32]	Lateral medulla [32]
AICA	Vestibular root entry zone [11, 12] Inner ear [11], flocculus (unilateral) [29], pontine tegmentum [12, 23]	Dorsal pontine tegmentum [14, 30], Flocculus (bilateral) [27]	MCP [33]	MCP [33]	Vestibular root entry zone [11, 12], Inner ear [11], MCP [33]
SCA	Superior vermis [13], rostral anterior cerebellum [13]	BC [26, 28]			
Basilar perf	NPH [12, 23], pontine tegmentum [11]	PMT [28], VTT [23, 28], MLF [26, 28]		PMT [28], VTT [23, 28], MLF [26, 28]	
Midbrain perf			INC [23, 31], riMLF [23, 31]	INC [23, 31], riMLF [23, 31]	

*AICA*  anterior inferior cerebellar artery, *BC*  brachial conjunctivum, *Horiz-tors*  Horizontal-Torsional, *ICP*  inferior cerebellar peduncle, *INC*  Interstitial nucleus of Cajal, *Lat*  lateral, *Med*  medial, *MCP*  middle cerebellar peduncle, *MLF*  medial longitudinal fasciulus, *NPH*  nucleus prepositus hypoglossi, *perf * perforators, *PICA*  posterior inferior cerebellar artery, *PMT*  paramedian tract, *riMLF*  rostral interstitial nucleus of medial longitudinal fasciculus, *SCA*  superior cerebellar artery, *VNc*  vestibular nucleus, *Vert-Tors*  Vertical-torsional, *VTT*  ventral tegmental tract

## Study limitations

The high rates of the absence of nystagmus recorded in PCS may be due to patients’ delayed presentation to ER. However, the prevalence of nystagmus in earlier reports of PCS was less than 100% (24–63%) [37–39]. Our AVN group whilst having diagnostic quantitative vestibular testing, did not all receive MRI imaging. Additionally, nystagmus could only be analysed in the horizontal and vertical plane using a monocular trace with torsional-plane traces unavailable. Positional nystagmus, which has value in the assessment of AVS [40], was inconsistently tested as patients were often too unwell to have those recordings done. Despite recruitment being at two international sites, further studies are required to enhance the generalizability of our findings to other population groups. Finally, multiple testing of statistical inferences on the same database was not controlled for in the analyses and some subgroup analysis was limited due to stroke numbers and diversity.

## Conclusions

Our study found that: (1) There was no single distinctive nystagmus pattern for PCS. (2) Given the paucity of nystagmus in some PCSs, we recommend a high index of suspicion for stroke in the symptomatic AVS patient who demonstrates *absence of nystagmus* with Frenzel glasses or fixation denied VNG. (3) The nystagmus recordings in a majority of AVN, in contrast, demonstrated unidirectional horizontal nystagmus. (4) The absence of typical “peripheral-appearing” nystagmus features should suggest PCS however the converse is not true: peripheral nystagmus does not imply a peripheral cause. (5) The presence of vascular risk factors can be useful in separating PCS from AVN. Thus, nystagmus recordings in the ER are a helpful and easy tool to assist the clinician in the diagnosis of PCS presenting with vertigo or imbalance.

## Supplementary Information

Below is the link to the electronic supplementary material.

Supplementary file1 (DOCX 17 KB)

Supplementary file2 (MP4 9958 KB)

Supplementary file3 (MP4 4148 KB)

Supplementary file4 (MP4 6013 KB)

Supplementary file5 (MP4 4745 KB)

Supplementary file6 (MP4 9008 KB)

Supplementary file7 (MP4 4653 KB)

Supplementary file8 (MP4 9935 KB)

Supplementary file9 (MP4 6234 KB)

Supplementary file10 (MP4 7131 KB)
