# Supplementary material for: Capturing nystagmus in the emergency room: posterior circulation stroke versus acute vestibular neuritis
Source: J Neurol. 2022 Jul 18;270(2):632–41. doi: 10.1007/s00415-022-11202-y (PMC9886594; doi:10.1007/s00415-022-11202-y)
Supplement: Supplementary file 1 — Supplementary file1 (DOCX 17 KB) [file 415_2022_11202_MOESM1_ESM.docx]

| **AVS aetiology** (n= 268) | **Nil nystagmus (%)** | **Horizontal nystagmus (n=)** | **Mean Horizontal SPV (^○^/s)** | **Vertical nystagmus (n=)** | **Mean vertical SPV (^○^/s)** | **Torsional nystagmus (n=)** |
| --- | --- | --- | --- | --- | --- | --- |
| **Vestibular neuritis** n=107 (39.9%) | 0 | 105 (98.1%) | 10.8±6.5 | 0 | 6.5* | 2 (1.9%) |
| **Posterior circulation stroke** n=93 (34.7%) | 31 (33.3%) | 46 (49.4%) | 5.1±3.1 | 10 (10.8%) | 6.2±3.9* | 6 (6.5%) |
| Radiologically confirmed n=73 (27.2%) | 26 (35.7%) | 36 (49.3%) | 4.9±2.9 | 8 (10.9%) | 6.9±4.4* | 3 (4.1%) |
| DWI negative n=20 (7.5%) | 5 (25%) | 10 (50%) | 5.9±3.7 | 2 (10%) | 4.6±1.8* | 3 (15%) |
| **Vestibular migraine** n=23 (8.6%) | 15 (62.5%) | 7 (30.4%) | 3.9±1.1 | 0 | 5.4* | 1 (4.3%) |
| **Other diagnoses** n=34 (12.7%)  Meniere’s n=5 (1.9%)  Vertigo and SSNHL n=5 (1.9%)  Demyelination n=5 (1.9%)  Other Central vertigo n=5 (1.9%)  Labyrinthitis n=3 (1.1%)  Ramsay-Hunt n=3 (1.1%)  Autoimmune inner ear disease n=2 (0.7%)  Iatrogenic/Trauma n=3 (1.1%)  Carcinomatosis meningitis n=1  Wernicke’s encephalopathy n=1  Vestibulopathy BPV canal jam n=1 | 1 (20%)  3 (60%)  2 (40%)  2 (40%)  0  0  0  0  0  1 (100%) | 4 (80%)  2 (40%)  3 (60%)  3 (60%)  3 (100%)  3 (100%)  1 (50%)  3 (100%)  1 (100%)  0 | 5.4±2.8  6.2  3.5±1.6  7.3±8.3  7.8±5.5  6.4±3.3  1.9  7.1±3.4  11.5  N/A | 0  0  0  0  0  0  1  0  0  0 | N/A  N/A  N/A  N/A  N/A  N/A  N/A**  N/A  N/A  N/A | 0  0  0  0  0  0  0  0  0  0 |
| **Unknown** n=11 (4.1%) | 5 (45.5%) | 6 (54.5%) | 6.4±4.4 | 0 | N/A | 0 |

**Supplementary Appendix Table 1: Acute vestibular neuritis aetiology and nystagmus features**

*****Vertical nystagmus SPV value calculated from those patients with pure vertical or vertical-torsional nystagmus

^**^ Video nystagmus was unanalysable

Abbreviations: AVS = acute vestibular syndrome, BPV=benign positional vertigo, DWI=diffusion weighted imaging, SPV = slow phase velocity, SSNHL = sudden sensorineural hearing loss
